# Treatment, Survival, and Prognosis of Advanced-Stage Natural Killer/T-Cell Lymphoma: An Analysis From the China Lymphoma Collaborative Group

**DOI:** 10.3389/fonc.2020.583050

**Published:** 2021-02-19

**Authors:** Weiping Liu, Yong Yang, Shunan Qi, Ying Wang, Xia He, Liling Zhang, Baolin Qu, Liting Qian, Xiaorong Hou, Xueying Qiao, Hua Wang, Gaofeng Li, Yujing Zhang, Yuan Zhu, Jianzhong Cao, Junxin Wu, Tao Wu, Suyu Zhu, Mei Shi, Liming Xu, Hang Su, Ningjing Lin, Jun Zhu, Yexiong Li, Yuqin Song

**Affiliations:** ^1^ Department of Radiation Oncology Key Laboratory of Carcinogenesis and Translational Research (Ministry of Education), Department of Lymphoma, Peking University Cancer Hospital & Institute, Beijing, China; ^2^ National Cancer Center/Cancer Hospital, Chinese Academy of Medical Sciences (CAMS) and Peking Union Medical College (PUMC), Center for Cancer Precision Medicine, CAMS and PUMC, National Institute of Biological Sciences, Collaborative Innovation Center for Cancer Medicine, Beijing, China; ^3^ Department of Radiation Oncology, Chongqing University Cancer Hospital & Chongqing Cancer Hospital, Chongqing, China; ^4^ Department of Radiation Oncology, The Affiliated Cancer Hospital of Nanjing Medical University and Jiangsu Cancer Hospital & Jiangsu Institute of Cancer Research, Nanjing, Jiangsu, China; ^5^ Cancer Center, Union Hospital, Tongji Medical College, Huazhong University of Science and Technology, Wuhan, China; ^6^ Department of Radiation Oncology, The First Medical Center of General Hospital of Chinese People’s Liberation Army, Beijing, China; ^7^ Department of Radiation Oncology, The Affiliated Provincial Hospital of Anhui Medical University, Hefei, China; ^8^ Department of Radiation Oncology, Peking Union Medical College Hospital, Chinese Academy of Medical Sciences (CAMS) and Peking Union Medical College (PUMC), Beijing, China; ^9^ Department of Radiation Oncology, The Fourth Hospital of Hebei Medical University, Shijiazhuang, China; ^10^ Department of Oncology, Second Affiliated Hospital of Nanchang University, Nanchang, China; ^11^ Department of Radiation Oncology, National Center of Gerontology, Institute of Geriatric Medicine, Beijing Hospital, Chinese Academy of Medical Sciences, Beijing, China; ^12^ Department of Radiation Oncology, Sun Yat-sen University Cancer Center, State Key Laboratory of Oncology in South China, Collaborative Innovation Center for Cancer Medicine, Guangzhou, China; ^13^ Department of Radiation Oncology, Zhejiang Cancer Hospital, Hangzhou, China; ^14^ Department of Radiation Oncology, Shanxi Cancer Hospital and the Affiliated Cancer Hospital of Shanxi Medical University, Taiyuan, China; ^15^ Department of Radiation Oncology, Fujian Provincial Cancer Hospital, Fuzhou, China; ^16^ Department of Radiation Oncology, Affiliated Hospital of Guizhou Medical University, Guizhou Cancer Hospital, Guiyang, China; ^17^ Department of Radiation Oncology, Hunan Cancer Hospital and the Affiliated Cancer Hospital of Xiangya School of Medicine, Changsha, China; ^18^ Department of Radiation Oncology, Xijing Hospital of Fourth Military Medical University, Xi’an, China; ^19^ Department of Radiation Oncology, Tianjin Medical University Cancer Institute & Hospital, Key Laboratory of Cancer Prevention and Therapy, National Clinical Research Center for Cancer, Tianjin, China; ^20^ Department of Lymphoma, The Fifth Medical Center of General Hospital of Chinese People’s Liberation Army, Beijing, China

**Keywords:** lymphoma, non-Hodgkin, lymphoma, extranodal NK-T-cell, therapeutics, prognosis, survival

## Abstract

Patients with advanced-stage natural killer/T-cell lymphoma (NKTCL) usually have a poor prognosis. However, there is limited data of comprehensive analysis on this particular patient population due to the rarity of the disease. The present study aimed to investigate the treatment models, survival outcomes, and prognosis of advanced-stage NKTCL. Data from 336 patients with advanced-stage NKTCL diagnosed between 2006 and 2015 in the China Lymphoma Collaborative Group database were retrospectively analyzed. The median age was 42 years and the male/female ratio was 2.4:1. About 97% of patients had stage IV disease and 77% had >1 extranodal involvement site. All patients received chemotherapy, with the most common option being asparaginase (Asp)-containing regimens (n=146; 43.5%). Among 286 patients with available response data, the overall response rate (ORR) was 57.3% with a complete remission (CR) rate of 35.7%. Asp-containing regimens led to better ORRs (86/132, 65.2% vs. 54/113, 47.8%, *P* = 0.006) and CR rates (60/132, 45.5% vs. 27/113, 23.9%, *P* < 0.001) than non-Asp-containing regimens. The expected 5-year progression-free survival (PFS) and overall survival (OS) rates were 22.6 and 32.0%, respectively, for the whole cohort. Compared to non-Asp-containing chemotherapy, Asp-containing chemotherapy improved 5-year PFS (34.2 vs. 17.1%, *P* < 0.001) and OS (45.3 vs. 27.8%, *P* < 0.001). A trend toward improvement in OS was observed when gemcitabine was added to Asp-containing chemotherapies. Moreover, those undergoing autologous hematopoietic stem cell transplantation had prolonged survival time. In conclusion, Asp-containing chemotherapy could improve the prognosis of advanced-stage NKTCL, and refinement of treatment models is warranted in the future.

## Introduction

Natural killer/T-cell lymphoma (NKTCL) is an unusual subtype of non-Hodgkin lymphoma (NHL) with a variable geographical distribution (1, 2). For example, the US only had an estimated 190 new cases of NKTCL in 2016, based on the incidence rate of 0.1 per 100,000 people during 2011–2012 (3). However, a population-based study suggested that NKTCL accounted for 1.2% of malignant lymphomas diagnosed in Japan, compared to only 0.2% of cases diagnosed in the US (4).

Disease status, including stage, helps to determine the prognosis of NKTCL. For example, patients with early-stage NKTCL have a favorable prognosis (5-year overall survival [OS] rate of approximately 70%) after receiving radiotherapy and asparaginase (Asp)-containing chemotherapies (5). In contrast, patients with advanced-stage NKTCL usually have a significantly inferior prognosis, based on 5-year OS rates of about 15–25% (6–8).

However, there have been no large-scale studies specifically focused on advanced-stage NKTCL due to the rarity of the disease. This study aimed to elucidate the clinical characteristics, treatment models, response rates, and survival outcomes for advanced-stage NKTCL.

## Methods

### Patients and Study Design

This study’s retrospective protocol was approved by the Ethics Committee of Peking University Cancer Hospital and Institute and the participating centers’ institutional review boards and complied with the Declaration of Helsinki. The requirement for informed consent was waived owing to the use of a deidentified data set.

A total of 348 patients with newly diagnosed advanced-stage NKTCL at 20 Chinese institutions between 2006 and 2015 were enrolled in the China Lymphoma Collaborative Group database. The eligibility criteria for the present study were: (a) histologically confirmed NKTCL according to the World Health Organization classification of neoplastic diseases of lymphoid tissues (9) and (b) stage III–IV disease according to the Ann-Arbor staging system (10, 11). Twelve patients were excluded, of which five were excluded due to incomplete data and seven due to palliative therapy. Thus, 336 patients were included in the analyses.

Clinical data such as age, sex, Eastern Cooperative Oncology Group performance status, B symptoms, extranodal involvement, and lactate dehydrogenase levels were collected. Given that Asp, including L-Asp and Peg-Asp, was used widely over time, chemotherapy regimens were divided into Asp-containing regimens and non-Asp-containing regimens. The nomogram-revised risk index (NRI) (12) was used for risk stratification.

### Statistics

Categorical variables were compared using Pearson’s χ^2^ analysis or Fisher’s exact test. The progression-free survival (PFS) interval was calculated from the diagnosis to the first instance of relapse or progression, last follow-up, or death because of any cause. The OS interval was calculated from the diagnosis to the first instance of the last follow-up or death because of any cause. Kaplan-Meier curves and the log-rank test were used to evaluate inter-group differences in PFS and OS. Propensity score-matched (PSM) analysis was then performed to test the survival benefits of chemotherapy regimens. All statistical analyses were performed using IBM SPSS software (version 21.0; IBM Corp., Armonk, NY, USA).

## Results

### Patient Characteristics

**Figure 1:**
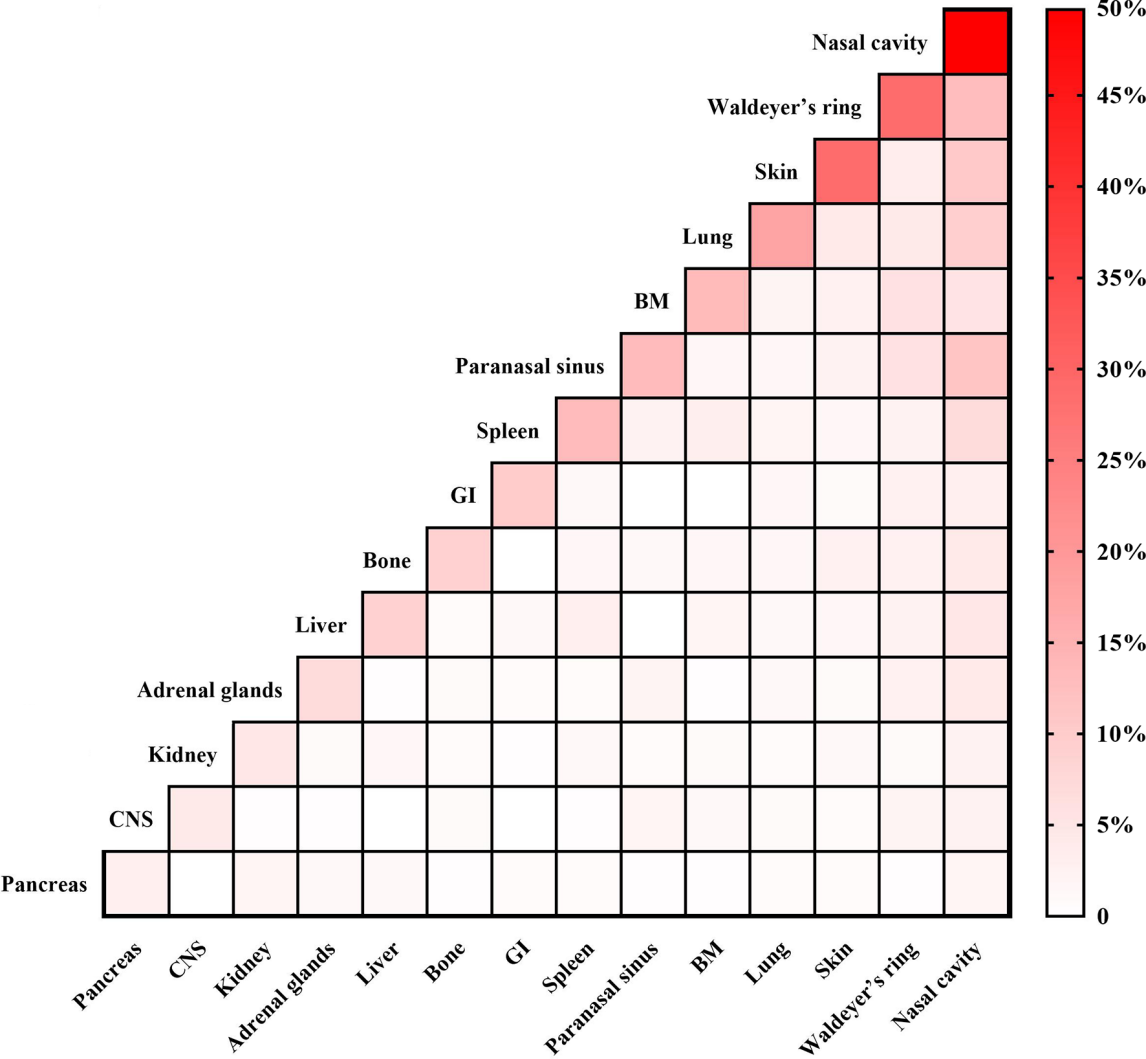
Distribution of extranodal involvement sites in 336 patients with advanced-stage natural killer/T-cell lymphoma. BM, bone marrow; CNS, central nervous system; GI, gastrointestinal tract.

The median age was 42 years (range: 6–84 years) and the male/female ratio was 2.4:1. As shown in **Table 1**, 96.7% of patients had stage IV disease, 76.5% had >1 extranodal involvement site, and 54.8% presented with B symptoms. As shown in **Figure 1**, the most frequent extranodal involvement site was the nasal cavity (49.7%), followed by Waldeyer’s ring (28.6%) and the skin (28.6%). Based on the NRI, about half of the patients were stratified into the very high-risk group. Those patients who received Asp-containing chemotherapy had comparable characteristics with those who received non-Asp-containing chemotherapy (**Table 1**).

**Table 1:** Baseline characteristics of 336 patients with advanced-stage natural killer/T-cell lymphoma.

Characteristic	Number (%)				P value*
	Total	Asp-containing regimen cohort	Non-Asp-containing regimen cohort	Unknown regimen cohort	
Sex					0.008
Male	238 (70.8)	109 (74.7)	73 (60.8)	56 (80.0)	
Female	98 (29.2)	37 (25.3)	47 (39.2)	14 (20.0)	
Age					0.690
≤60 years	297 (88.4)	131 (89.7)	106 (88.3)	60 (85.7)	
>60 years	39 (11.6)	15 (10.3)	14 (11.7)	10 (14.3)	
Stage					0.141
III	11 (3.3)	3 (2.1)	7 (5.8)	1 (1.4)	
IV	325 (96.7)	143 (97.9)	113 (94.2)	69 (98.6)	
ECOG PS					0.178
0–1	260 (77.4)	120 (82.2)	89 (74.2)	51 (72.9)	
2–4	76 (22.6)	26 (17.8)	31 (25.8)	19 (27.1)	
Elevated LDH					0.337
Yes	178 (53.0)	72 (49.3)	64 (53.3)	42 (60.0)	
No	158 (47.0)	74 (50.7)	56 (46.7)	28 (40.0)	
B symptoms					0.889
Yes	184 (54.8)	82 (56.2)	65 (54.2)	37 (52.9)	
No	152 (45.2)	64 (43.8)	55 (45.8)	33 (47.1)	
Extranodal involvement >1					0.032
Yes	257 (76.5)	121 (82.9)	83 (69.2)	53 (75.7)	
No	79 (23.5)	25 (17.1)	37 (30.8)	17 (24.3)	
NRI risk stratification					0.187
Low	0 (0)	0 (0)	0 (0)	0 (0)	
Intermediate high	45 (13.4)	21 (14.4)	20 (16.7)	4 (5.7)	
High	129 (38.4)	53 (36.3)	49 (40.8)	27 (38.6)	
Very high	162 (48.2)	72 (49.3)	51 (42.5)	39 (55.7)	

ECOG PS, Eastern Clinical Oncology Group performance status; LDH, lactate dehydrogenase; NRI, nomogram-revised risk index.

*Comparison of the difference among the Asp-containing, non-Asp containing, and unknown regimen cohorts.

### Treatment Model and Response Rates

All patients received primary treatment using chemotherapy regimens; of them, 146 (43.5%) patients were treated with Asp-containing regimens, 120 (35.7%) with non-Asp-containing regimens, and 70 (20.8%) with unknown regimens (**Appendix Table S1**). In addition, radiotherapy was administered in 14 (4.2%) patients. Thirteen patients also underwent autologous hematopoietic stem-cell transplantation (AHSCT) after their first remission.

Data regarding treatment responses were available for 286 patients. The overall response rate (ORR) was 57.3%, with complete response (CR) achieved by 102 patients (35.7%) and partial response (PR) achieved by 62 patients (21.7%). Compared with non-Asp-Containing chemotherapy, Asp-containing chemotherapy provided better response rates (ORR, 65.2 vs. 47.8%, *P* = 0.006; CR, 45.5 vs. 23.9%, *P* < 0.001). Among patients treated with Asp-containing chemotherapy, those who also received gemcitabine had higher response rates than those who did not receive gemcitabine (ORR, 81.5 vs. 53.8%, *P* = 0.001; CR, 55.6 vs. 38.5%, *P* = 0.052).

### Survival Outcome and Prognosis

During the median follow-up period of 33.6 months (range: 0.3–128.8 months), 185 patients (64.7%) experienced disease progression, and 152 patients (53.1%) died. In the entire cohort, the expected 5-year PFS and OS rates were 22.6 and 32.0%, respectively.

**Figure 2:**
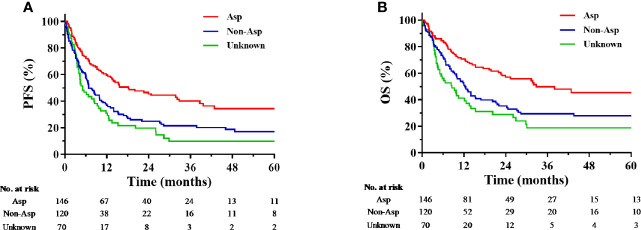
Kaplan-Meier progression-free survival **(A)** and overall survival **(B)** curves for the subgroups receiving chemotherapy with different regimens. Asp, asparaginase-containing regimens; Non-Asp, non-asparaginase-containing regimens; OS, overall survival; PFS, progression-free survival.

**Figure 3:**
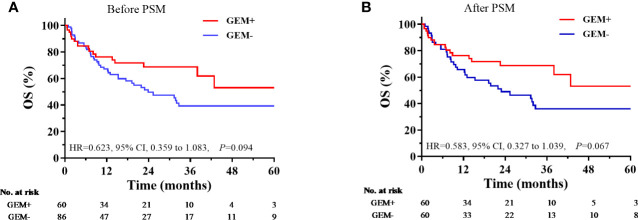
Comparison of overall survival according to the use of asparaginase-containing chemotherapy with and without gemcitabine. **(A)** Before PSM, **(B)** After PSM. CI, confidence interval; GEM+, with gemcitabine; GEM-, without gemcitabine; HR, hazard ratio; OS, overall survival; PSM, propensity score matching.

Compared with non-Asp-containing chemotherapy, Asp-containing chemotherapy improved survival outcome (5-year PFS, 34.2 vs. 17.1%, *P* < 0.001, **Figure 2A**; 5-year OS, 45.3 vs. 27.8%, *P* < 0.001, **Figure 2B**). A trend toward improvement in OS was observed when gemcitabine was added to Asp-containing chemotherapy (**Figure 3**).

Among the 13 patients who received AHSCT, 12 patients remained in remission with a median OS interval of 43.0 months (range: 13.3–82.5 months), and 1 patient died because of lymphoma progression at 7 months after AHSCT (survival time of 17.5 months) (**Appendix Table S2**).

According to the NRI, the 5-year OS in the intermediate high-, high-, and very high-risk groups were 51.2%, 37.9%, and 24.8% for the entire cohort and 63.2%, 48.8%, and 37.3% for those patients who received Asp-containing chemotherapy, respectively.

## Discussion

Patients with advanced-stage NKTCL were usually considered to have a poor prognosis. However, there was no large-scale study focusing on this particular patient population, especially in the era of Asp-containing chemotherapy. Using a cohort of 336 cases, we determined clinical characteristics, indicated the survival benefit provided by Asp-containing chemotherapy, and explored the heterogeneous prognosis of advanced-stage NKTCL.

During the last decade, chemotherapy for NKTCL has evolved from anthracycline-containing regimens to Asp-containing regimens, such as the SMILE regimen (dexamethasone, methotrexate, ifosfamide, L- Asp, and etoposide) (13). A phase II study involving 38 patients with newly diagnosed stage IV or relapsed/refractory disease revealed that the SMILE regimen provided good efficacy based on an ORR of 79% and a CR rate of 40% (14). However, another study revealed that SMILE-based chemotherapy provided unfavorable efficacy based on an ORR of 59%, severe toxicity (treatment-related mortality: 18.5%), and poor survival outcomes (median OS: 10.6 months) (15). A prospective study of 42 patients with stage III–IV disease also revealed that the DDGP regimen (cisplatin, dexamethasone, gemcitabine, and Peg- Asp) might be superior to the SMILE regimen based on the ORRs (95% vs. 67%) and the CR rate (71% vs. 29%) (16). Nevertheless, selection bias and the limited sample sizes might have influenced the treatment response rates and survival outcomes (17). In the present large-scale study, we found that Asp-containing regimens enhanced the efficacy of chemotherapy with an increase of 20% in ORR and CR rates, and it provided an additional survival benefit with an approximately 15% increase in the 5-year PFS and 5-year OS rates compared to the non-Asp-containing chemotherapies. Moreover, there was a trend toward improvement in survival outcomes when gemcitabine was added to Asp-containing chemotherapy. These findings indicated the survival benefits provided by Asp-containing chemotherapy and suggested the potential refining of induction chemotherapy by the inclusion of gemcitabine.

The use of AHSCT as a front-line consolidation treatment helped to improve outcomes in cases of advanced-stage NKTCL, which agrees with recommendations from the clinical practice guidelines (18). A retrospective study of 47 patients with NKTCL and 107 historical controls revealed median survival times of not reached in the AHSCT group and 43.5 months in the control group (19). Moreover, among patients who achieved CR, AHSCT was associated with a significantly increased 5-year OS rate (87.3% vs. 67.8%). A prospective study involving 27 patients with stage IV NKTCL showed that the consolidation with AHSCT resulted in a higher percentage (63% vs. 40%) of patients who were alive during the median follow-up period of 28.4 months (15). The present study only included a small proportion of patients who underwent AHSCT (3.4%), which makes it difficult to definitively comment on the effects of this treatment for advanced-stage NKTCL. Nevertheless, long-term survival without disease progression was observed in 12 of the 13 patients who underwent AHSCT. These findings implied that consolidative AHSCT should be considered for advanced-stage NKTCL and especially for patients who have achieved remission.

In a previous study (12), the NRI was proposed for risk stratification in cases of NKTCL. The NRI was calculated using the following five risk factors: age >60 years (1 point), Eastern Clinical Oncology Group performance status ≥2 (1 point), elevated lactate dehydrogenase levels (1 point), primary tumor invasion (1 point), and stage II (1 point) or stage III-IV disease (2 points). Patients were stratified into low-, intermediate low-, intermediate high-, high- and very high-risk groups when they scored 0, 1, 2, 3, and ≥4 points according to those risk factors, respectively. The NRI has been proven to provide good discrimination in the prognosis of NKTCL, better than that provided by other prognostic indexes such as the International Prognostic Index (20) and prognostic index of natural killer lymphoma (21). In the present study, NRI could stratify the 5-year OS rates of advanced-stage NKTCL ranging from 24.8% to 51.2% with approximately 10–15% differences between each risk group, and its prognostic value remained robust in subgroups of patients who received Asp-containing chemotherapy.

The present study has several limitations. First, the retrospective design did not involve a central pathological review of the biopsy samples, although the reviews were performed by experienced pathologists at tertiary hospitals. Second, although Asp-containing chemotherapies provide better survival outcomes than non-Asp-containing chemotherapies, the heterogeneous regimens used in the present study make it difficult to identify a single optimal regimen. Third, we did not have access to data regarding potentially relevant biomarkers, such as the pre-treatment Epstein-Barr virus DNA load (21) or soluble interleukin-2 concentrations (7).

In conclusion, the present study evaluated a large series of patients with advanced-stage NKTCL who received treatment in real-world settings. The NRI demonstrated good discriminative ability for prognosis, which remained applicable to patients who received modern Asp-containing regimens. Controlling for the selection biases by PSM analysis, our results suggested that Asp-containing chemotherapy enhanced treatment efficacy and provided survival benefits. In the future, the refinement of treatment models is warranted.

## Data Availability Statement

The raw data supporting the conclusions of this article will be made available by the authors, without undue reservation.

## Ethics Statement

This study’s retrospective protocol was approved by the Ethics Committee at Peking University Cancer Hospital and Institute and the participating centers’ institutional review boards and complied with the Declaration of Helsinki. The requirement for informed consent was waived based on the use of a deidentified data set.

## Author Contributions

WL, YY, and SQ conceived and designed the study, analyzed the data, and drafted and revised the paper. All authors collected the data and provided critical comments on the manuscript. YS and LN drafted and revised the paper. YL and YS designed the study, interpreted the results, and drafted and revised the paper. All authors contributed to the article and approved the submitted version.

## Funding

This study was supported by the Capital’s Funds for Health Improvement and Research of China (grant no. 2018-1-2151). The funders of the study had no role in study design, data collection, data analysis, data interpretation, or writing of the report.

## Conflict of Interest

The authors declare that the research was conducted in the absence of any commercial or financial relationships that could be construed as a potential conflict of interest.
