# Supplementary material for: Treatment, Survival, and Prognosis of Advanced-Stage Natural Killer/T-Cell Lymphoma: An Analysis From the China Lymphoma Collaborative Group
Source: Front Oncol. 2021 Feb 19;10:583050. doi: 10.3389/fonc.2020.583050 (PMC7945040; doi:10.3389/fonc.2020.583050)
Supplement: Supplementary file 1 [file DataSheet_1.docx]

Table S1. Distribution of chemotherapy regimens in 336 patients with advanced-stage NKTCL

|  | Number (%) | Definition and agents |
| --- | --- | --- |
| Asp–containing | 146 (43.5) | Regimens containing asparaginase (L-asparaginase or Peg-asparaginase) |
| GEMOX-L/P | 26 (7.7) | Gemcitabine, oxaliplatin, L-asparaginase or Peg-asparaginase |
| GDP-L/P | 11 (3.3) | Gemcitabine, cisplatin, dexamethasone, L-asparaginase or Peg-asparaginase |
| DDGP | 3 (0.9) | Cisplatin, dexamethasone, gemcitabine, Peg-asparaginase |
| GDP/VIDL | 1 (0.3) | Part A, Gemcitabine, cisplatin, dexamethasone; Part B, etoposide, ifosfamide, dexamethasone, L-asparaginase |
| IPGDP | 1 (0.3) | Ifosfamide, Peg-asparaginase, gemcitabine, cisplatin, dexamethasone |
| GEMOX-L/P-VP16 | 1 (0.3) | Gemcitabine, oxaliplatin, L-asparaginase or Peg-asparaginase, etoposide |
| GDLE | 1 (0.3) | Gemcitabine, cisplatin, L-asparaginase, etoposide |
| MICE-L | 1 (0.3) | Methotrexate, ifosfamide, carboplatin, etoposide, L-asparaginase |
| CHOP-L/P | 35 (10.4) | Cyclophosphamide, doxorubicin, vincristine, prednisone, L-asparaginase or Peg-asparaginase |
| COEP-L/P | 14 (3.6) | Cyclophosphamide, vincristine, etoposide, prednisone, L-asparaginase or Peg-asparaginase |
| AspaMetDex-GEM | 14 (4.2) | L-asparaginase, methotrexate, dexamethasone, gemcitabine |
| LOP | 10 (3.0) | L-asparaginase, vincristine, prednisone |
| AspaMetDex | 6 (1.6) | L-asparaginase, methotrexate, dexamethasone |
| SMILE | 6 (1.8) | Dexamethasone, methotrexate, ifosfamide, L-asparaginase, etoposide |
| EPOCH/VIDL | 5 (1.5) | Part A, etoposide, prednisone, vincristine, cyclophosphamide, doxorubicin; Part B, etoposide, ifosfamide, dexamethasone, L-asparaginase |
| MESA | 3 (0.9) | Methotrexate, etoposide, dexamethasone, Peg-asparaginase |
| IGE-P | 2 (0.6) | Ifosfamide, gemcitabine, etoposide, Peg-asparaginase |
| EPOCH-L/P | 2 (0.6) | Etoposide, prednisone, vincristine, cyclophosphamide, doxorubicin, L-asparaginase or Peg-asparaginase |
| VDLP | 2 (0.6) | Vincristine, daunorubicin, L-asparaginase, prednisone |
| CHOEP-L/P | 2 (0.6) | Cyclophosphamide, doxorubicin, vincristine, etoposide, prednisone, L-asparaginase or Peg-asparaginase |
| Non-Asp–containing | 120 (35.7) | Regimens without asparaginase (L-asparaginase or Peg-asparaginase) |
| CHOP | 62 (18.5) | Cyclophosphamide, doxorubicin, vincristine, prednisone |
| CHOEP | 13 (3.9) | Cyclophosphamide, doxorubicin, vincristine, etoposide, prednisone |
| EPOCH | 15 (4.5) | Etoposide, prednisone, vincristine, cyclophosphamide, doxorubicin |
| BACOP | 0 (3.1) | Bleomycin, doxorubicin, cyclophosphamide, vincristine, prednisone |
| CHOP-BLM | 3 (0.9) | Cyclophosphamide, doxorubicin, vincristine, prednisone, bleomycin |
| ProMACE-CytaBOM | 1 (0.3) | Cyclophosphamide, vincristine, doxorubicin, prednisone, cytarabine, etoposide, bleomycin, methotrexate |
| CHOP-VM-26 | 2 (0.6) | Cyclophosphamide, doxorubicin, vincristine, prednisone, teniposide |
| Hyper CVAD | 1 (0.3) | Part A, cyclophosphamide, doxorubicin, vincristine, dexamethasone, Part B, methotrexate, cytarabine |
| Others | 23 (6.8) | Regimens without inclusion of regimens previously listed |
| GDP | 6 (1.8) | Gemcitabine, cisplatin, dexamethasone |
| VIPD | 6 (1.8) | Etoposide, ifosfamide, cisplatin, dexamethasone |
| DICE | 4 (1.2) | Dexamethasone, ifosfamide, carboplatin, etoposide |
| CVP | 2 (1.0) | Cyclophosphamide, vincristine, prednisone |
| GEMOX | 2 (0.6) | Gemcitabine, oxaliplatin |
| IMVP16 | 1 (0.3) | Ifosfamide, methotrexate, etoposide |
| ICE | 1 (0.3) | Ifosfamide, carboplatin, etoposide |
| MOP | 1 (0.3) | Methotrexate, vincristine, prednisone |
| Unknown | 70 (20.8) | Regimens unknown |

Abbreviations: Ant, anthracyclines; BLM, bleomycin; GEM, gemcitabine; Asp, asparaginase; NKTCL, natural killer/T-cell lymphoma; VM-26, teniposide; VP-16, etoposide

Table S2. The clinical characteristics and outcome of 13 patients underwent AHSCT

| Case | Age/Sex | Before AHSCT | | | After AHSCT | | PFS  (months) | OS  (months) |
| --- | --- | --- | --- | --- | --- | --- | --- | --- |
|  |  | Asp-based CT | RT | Response | Progression | Outcome |  |  |
| 1 | 56/F | No | No | CR | No | Alive | 82.5 | 82.5 |
| 2 | 42/M | Yes | No | PR | No | Alive | 68.7 | 68.7 |
| 3 | 41/M | Yes | No | CR | No | Alive | 81.4 | 81.4 |
| 4 | 52/M | Yes | Yes | CR | No | Alive | 64.2 | 64.2 |
| 5 | 19/M | Yes | No | CR | No | Alive | 59.6 | 59.6 |
| 6 | 19/M | Yes | No | CR | Yes | Died | 13.0 | 17.5 |
| 7 | 49/F | Yes | No | CR | No | Alive | 29.6 | 29.6 |
| 8 | 25/M | Yes | No | CR | No | Alive | 24.8 | 24.8 |
| 9 | 41/M | Yes | No | CR | No | Alive | 20.6 | 20.6 |
| 10 | 32/M | Yes | Yes | CR | No | Alive | 18.7 | 18.7 |
| 11 | 36/F | Yes | No | CR | No | Alive | 13.3 | 13.3 |
| 12 | 39/M | Yes | Yes | CR | No | Alive | 43.0 | 43.0 |
| 13 | 50/F | No | Yes | CR | No | Alive | 47.2 | 47.2 |

Abbreviations: AHSCT, autologous hematopoietic stem cell transplantation; Asp, asparaginase; CR, complete remission; CT, chemotherapy;

F, female; M, male; OS, overall survival; PFS, progression-free survival; PR, partial remission; RT, radiotherapy
